# A Machine Learning-Based Prediction Model for Acute Kidney Injury in Patients With Congestive Heart Failure

**DOI:** 10.3389/fcvm.2022.842873

**Published:** 2022-03-04

**Authors:** Xi Peng, Le Li, Xinyu Wang, Huiping Zhang

**Affiliations:** ^1^Department of Cardiology, National Center of Gerontology, Beijing Hospital, Beijing, China; ^2^Institute of Geriatric Medicine, Chinese Academy of Medical Sciences, Beijing, China; ^3^National Center for Cardiovascular Diseases, Peking Union Medical College, Fuwai Hospital, Chinese Academy of Medical Sciences, Beijing, China; ^4^Department of Environmental and Engineering, Beijing University of Civil Engineering and Architecture, Beijing, China

**Keywords:** acute kidney injury, congestive heart failure, prediction model, machine learning, LightGBM

## Abstract

**Background:**

Machine learning (ML) has been used to build high performance prediction model. Patients with congestive heart failure (CHF) are vulnerable to acute kidney injury (AKI) which makes treatment difficult. We aimed to establish an ML-based prediction model for the early identification of AKI in patients with CHF.

**Methods:**

Patients data were extracted from the Medical Information Mart for Intensive Care III (MIMIC-III) database, and patients with CHF were selected. Comparisons between several common ML classifiers were conducted to select the best prediction model. Recursive feature elimination (RFE) was used to select important prediction features. The model was improved using hyperparameters optimization (HPO). The final model was validated using an external validation set from the eICU Collaborative Research Database. The area under the receiver operating characteristic curve (AUROC), accuracy, calibration curve and decision curve analysis were used to evaluate prediction performance. Additionally, the final model was used to predict renal replacement therapy (RRT) requirement and to assess the short-term prognosis of patients with CHF. Finally, a software program was developed based on the selected features, which could intuitively report the probability of AKI.

**Results:**

A total of 8,580 patients with CHF were included, among whom 2,364 were diagnosed with AKI. The LightGBM model showed the best prediction performance (AUROC = 0.803) among the 13 ML-based models. After RFE and HPO, the final model was established with 18 features including serum creatinine (SCr), blood urea nitrogen (BUN) and urine output (UO). The prediction performance of LightGBM was better than that of measuring SCr, UO or SCr combined with UO (AUROCs: 0.809, 0.703, 0.560 and 0.714, respectively). Additionally, the final model could accurately predict RRT requirement in patients with (AUROC = 0.954). Moreover, the participants were divided into high- and low-risk groups for AKI, and the 90-day mortality in the high-risk group was significantly higher than that in the low-risk group (log-rank *p* < 0.001). Finally, external validation using the eICU database comprising 9,749 patients with CHF revealed satisfactory prediction outcomes (AUROC = 0.816).

**Conclusion:**

A prediction model for AKI in patients with CHF was established based on LightGBM, and the prediction performance of this model was better than that of other models. This model may help in predicting RRT requirement and in identifying the population with poor prognosis among patients with CHF.

## Introduction

Acute kidney injury (AKI) is a condition characterized by a rapid increase in serum creatinine (SCr), a decrease in urine output (UO) or both symptoms occurring simultaneously, accompanied by major complications including volume overload, electrolyte disorders, uremic complications, and drug toxicity (1). The incidence of AKI is 10–15% in patients admitted to the hospital (2), and is more than 50% in those in the intensive care unit (ICU) (3). Previous studies have demonstrated that even mild forms of AKI are strongly associated with poor prognosis (4, 5). In other words, patients who develop AKI have an increased risk of mortality. Moreover, there is a lack of effective treatment options for AKI, which leads to adverse outcomes for patients. Although renal replacement therapy (RRT) is a key treatment for advanced AKI, it also has the potential to cause some harm and is not available in certain settings and regions (6). Therefore, it is important to prevent AKI in hospitalized patients.

Although AKI is associated with many conditions, in most cases, it can be attributed to certain simple and common causes, including insufficient effective circulating volume (ECV) and hypotension (7). Accordingly, in the past decade, attention has shifted from treatment to prevention and early detection. For many years, increased SCr and decreased UO have been used to identify AKI in the short term (8). However, some issues including unsatisfactory accuracy, lack of specificity and hysteretic nature, limit the value of these measurements in the early detection of AKI (9, 10).

The relationship between heart failure and renal dysfunction is very complicated. Briefly, congestive heart failure (CHF) causes low ECV and reduces renal perfusion, which in turn increases the absorption of sodium-water, leading to a heavy load on the heart, thereby making the combination of these conditions extremely difficult to address (11). Because of the poor prognosis of AKI in patients with CHF (12), clinicians should pay more attention to identifying early renal dysfunction in such patients. Unfortunately, few studies were focused on this issue. Therefore, in the present study, we aimed to establish a prediction model for AKI in patients with CHF based on machine learning (ML).

## Materials and Methods

### Sources of Data

Medical Information Mart for Intensive Care III (MIMIC-III, version 1.4) is a large single-center database containing the medical records of ~60,000 ICU patients admitted to the Beth Israel Deaconess Medical Center (Boston, MA, USA) between 2001 and 2012 (13). To establish the prediction model, the data from the MIMIC-III database were split into a training set and an internal validation set. The eICU Collaborative Research Database (eICU, version 2.0) is a multicenter ICU database comparising high granularity data related to over 200,000 ICU admissions between 2014 and 2015 at 208 hospitals located throughout the United States (14). Data from the eICU database were used as an external validation set.

Researchers who completed and passed an online course on “Protecting Human Research Subjects” organized by the National Institutes of Health (NIH) are qualified to inquie about the information from the databases. One of the authors (LL) obtained the qualification (record ID: 35965741) and was responsible for data extraction. The Massachusetts Institute of Technology has approved the establishment of the databases with an informed consent exemption. The study was reported according to the recommendations of the Transparent Reporting of a multivariable prediction model for Individual Prognosis or Diagnosis (TRIPOD) statement (15).

### Study Population

Patients aged >18 years with CHF as the major cause of hospital admission were included in this retrospective cohort study. Patients with incomplete data were excluded from the study. In the two databases, CHF was diagnosed by clinicians based on the guideline of heart failure (16). We aimed to build a prediction model for AKI diagnosed based on the following clinical practice guidelines: increase in SCr by ≥0.3 mg/dL (or ≥ 26.5 μmol/L) in 48 h, increase in SCr to 1.5 times over baseline levels in 7 days, and patient UO ≤ 0.5 mL/kg/h for 6 h (8).

### Data Collection

We used PostgreSQL tools version 13.0, to extract medical data from the two databases. There were some patients had more than one ICU admission, the first data records (mostly within the first 24-h) in the first ICU admission were used in the analysis. Subject IDs were used to identify distinct patients. Data, such as the demographics, vital signs, common comorbidities, and laboratory tests results were included in the initial analysis.

### Development of the Prediction Model

In the present study, ML-based models were used to build a prediction model for AKI developing during the period of hospital admission in patients with CHF.

First, data from the MIMIC-III database were randomly split into the training set (80%) and internal validation set (20%). Models based on common ML classifiers including LightGBM, XGBoost, AdaBoost, CatBoost, gradient boosting decision tree (GBDT), bootstrap aggregating (Bagging), decision tree, random forest, logistic regression (LR), support vector machine (SVM), naïve Bayes, multi-layer perceptron neural networks (MLP) and k nearest neighbors (KNN) models were selected for making the initial prediction based on 58 features, and the model with the highest prediction value was selected as the primary model in this study.

Second, the recursive feature elimination (RFE) algorithm based on Shapley Additive explanations (SHAP) values was performed to identify key features, which helped in making the model more feasible for clinical practice. The effects of the remaining features on the prediction scores were then measured using the functions of the SHAP Python package (version 0.40.0), which assessed the importance of each feature using a game-theoretic approach (17). The feature with the smallest effect on the prediction was eliminated in each loop (18), and, a compact model was generated.

Third, hyperparameter optimization (HPO) was performed to improve the prediction performance of the selected model. For performing the optimization through the hyperband method and for testing different combinations of hyperparameters, we used Optuna version 2.10.0 (19), which is an open-source optimization framework that enables users to design complex deep learning experiments quickly, efficiently, and dynamically (20). A total of 100 trials were conducted, and the parameters with the greatest area under the receiver operating characteristic curve (AUROC) were saved.

**Figure 1:**
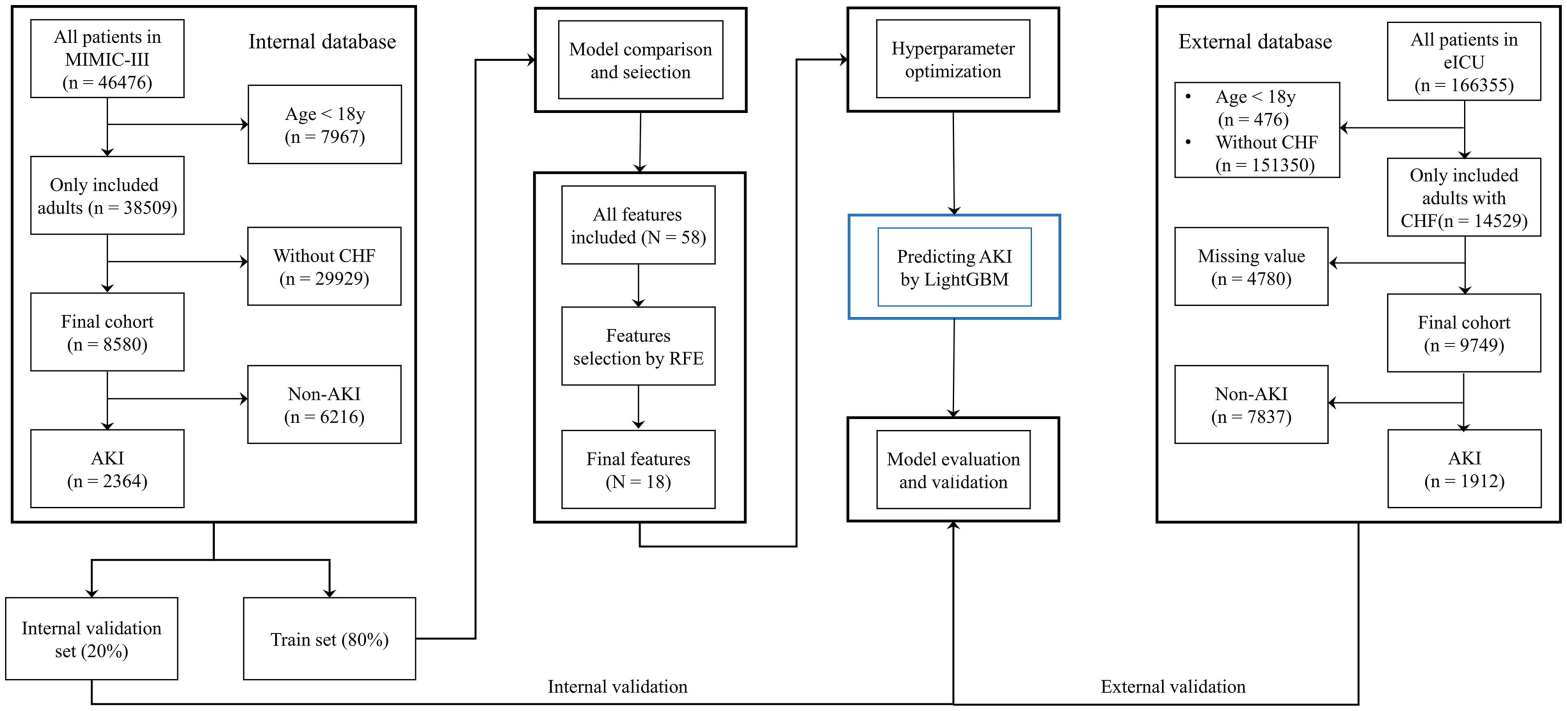
Flow chart.

Last, the final model was used to predict AKI based on the best combination of hyperparameters. Data from the eICU database were used as an external validation set to verify the model's value. To further demonstrate the performance of the prediction model, patients in the internal validation set were divided into high- and low-risk groups based on whether their AKI risk predicted by the final model was greater than the median risk in the set, and 30-day mortality was also compared between the two groups. Moreover, the requirement for RRT within the first 24-h after ICU admission was predicted based on the final model (Figure 1). In contrast to other studies involving prediction models, we attempted to develop a software program for calculating the possibility of AKI development in patients with CHF, which could help clinicians in easily identifying the high-risk patients and implementing effective prevention strategies.

### Statistical Analyses

The Kolmogorov-Smirnov test was used to evaluate the normal distribution of the data. Continuous variables were expressed as mean ± standard deviation (SD) and compared using *t*–tests. Levene's homogeneity of variance test was used to test the assumption of homoscedasticity. If the homoscedasticity was unsatisfactory, Welch's *t*-test was used for performing comparisons between the groups. Categorical data were expressed as proportions and were compared using the chi-squared test. The discriminative ability of the model in predicting AKI was assessed using AUROC. To further elucidate the performance of the model, calibration plotting and decision curve analysis (DCA) were performed. In addition, the accuracy, positive prediction value (PPV), negative prediction value (NPV), balanced accuracy (BA), F1-score and Matthews correlation coefficient (MCC) were calculated for each model in order to evaluate the model's value.

Both the MIMIC-III and eICU databases included extreme and missing values. Extreme and error values that failed the logic check were censored and were replaced with mean values. Variables with a missing values rate of more than 30% of the sample size were excluded. Mean imputation was performed to fill in missing data of <5%. Multiple imputation was used to handle variables with missing data of between 5 and 30% (21).

All statistical analyses were conducted using Python version 3.9.0 (Python Software Foundation, www.python.org) and Stata version 15.0 (StataCorp, College Station, TX, USA). A two-tailed test was performed, and a *P* < 0.05 was considered to reflect statistical significance.

## Results

### Baseline Characteristics

A total of 8,580 patients with CHF from the MIMIC-III database were finally included in the analysis. Among them, 2,364 patients were diagnosed with AKI. Additionally, 58 features, including patient demographics and characteristics, vital signs, therapy administered, comorbidities and laboratory values were used to build the initial model (Supplementary Table 1). After the feature selection process, 18 important features including age, weight, temperature, heart rate (HR), mean aortic pressure (MAP), UO within the first 24 h, partial arterial oxygen pressure (PaO_2_), arterial partial pressure of carbon dioxide (PaCO_2_), white blood cell count (WBC), red blood cell count (RBC), hematocrit, platelet count (PLT), SCr, blood urine nitrogen (BUN), creatine kinase (CK), blood lactate, blood glucose, and calcium were identified for establishing a compact model and for performing external validation using the eICU database. Comparisons between the non-AKI and AKI groups from the MIMIC-III and eICU databases are shown in Table 1. The first records of the abovementioned medical data were selected for analysis.

**Table 1:** Baseline characteristic of the cohorts.

	**MIMIC-III cohort (*n* = 8580)**			**eICU cohort (*n* = 9749)**		
**Variables**	**Non-AKI (*n* = 6,216)**	**AKI** **(*n* = 2,364)**	***P* value**	**Non-AKI** **(*n* = 7,837)**	**AKI (*n* = 1,912)**	***P* value**
**Demographics and vital signs**						
Age, years	72.3 ± 13.7	75.0 ± 12.7	<0.001	69.9 ± 13.9	70.5 ± 13.7	0.110
Weight, kg	80.6 ± 22.3	82.6 ± 24.8	<0.001	88.3 ± 29.5	91.7 ± 31.0	<0.001
Temperature, °C	36.8 ± 0.6	36.6 ± 0.7	<0.001	36.8 ± 0.9	36.4 ± 0.6	<0.001
HR, bpm	102.9 ± 21.2	102.1 ± 22.6	0.145	91.4 ± 18.4	92.1 ± 21.0	0.183
MAP, mmHg	75.7 ± 10.5	73.8 ± 10.8	<0.001	82.7 ± 20.2	75.9 ± 21.4	<0.001
UO, mL	1,813 ± 1,171	1,639 ± 1,193	<0.001	1,734 ± 1,539	1,226 ± 1,033	<0.001
**Laboratory tests**						
PaO_2_, mmHg	169.2 ± 88.7	137.7 ± 74.7	<0.001	119.4 ± 64.9	122.1 ± 72.2	0.112
PaCO_2_, mmHg	44.5 ± 12.7	44.1 ± 13.4	0.247	45.3 ± 16.5	40.8 ± 16.2	<0.001
WBC, ×10^9^/*L*	12.2 ± 6.1	12.2 ± 6.5	0.618	11.2 ± 5.6	12.4 ± 6.5	<0.001
RBC, ×10^1^2/*L*	3.57 ± 0.68	3.55 ± 0.70	0.138	4.00 ± 0.81	3.86 ± 0.89	<0.001
Hematocrit, %	32.0 ± 6.1	31.9 ± 6.1	0.228	36.2 ± 7.1	34.7 ± 7.8	<0.001
Platelet, ×10^9^/*L*	216.2 ± 100.2	221.9 ± 105.1	0.024	228.4 ± 93.9	217.7 ± 97.7	<0.001
SCr, mg/dL	1.47 ± 1.55	2.04 ± 1.33	<0.001	1.90 ± 2.05	2.76 ± 2.19	<0.001
BUN, mg/dL	28.0 ± 19.9	46.1 ± 26.7	<0.001	31.3 ± 20.3	50.3 ± 29.0	<0.001
CK, U/L	467 ± 900	442 ± 926	0.246	245 ± 266	427 ± 474	<0.001
Lactate, mmol/L	2.21 ± 1.52	2.22 ± 1.62	0.832	2.31 ± 1.47	2.80 ± 2.13	<0.001
Glucose, mg/dL	141.8 ± 44.2	145.8 ± 48.8	<0.001	165.7 ± 89.6	169.3 ± 98.9	0.145
Calcium, mmol/L	2.10 ± 0.21	2.09 ± 0.22	0.542	2.06 ± 0.50	2.04 ± 0.48	0.091

*AKI, acute kidney injury; HR, heart rate; MAP, mean aortic pressure; UO, urine output; PaO_2_, partial arterial oxygen pressure; PaCO_2_, arterial partial pressure of carbon dioxide; WBC, white blood cell count; RBC, red blood cell count; SCr, Serum creatinine; BUN, blood urine nitrogen; CK, creatine kinase*.

### Development of LightGBM Model

**Figure 2:**
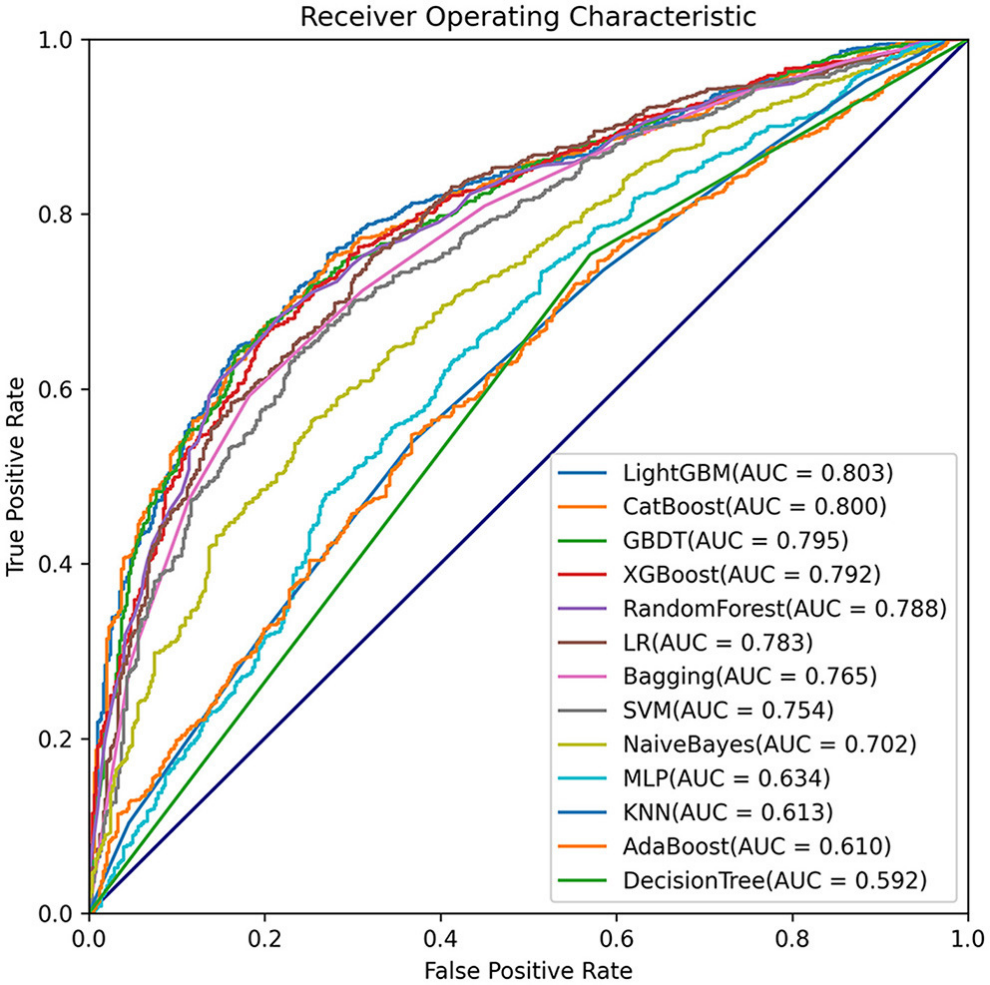
Comparisons of different machine learning models.

**Figure 3:**
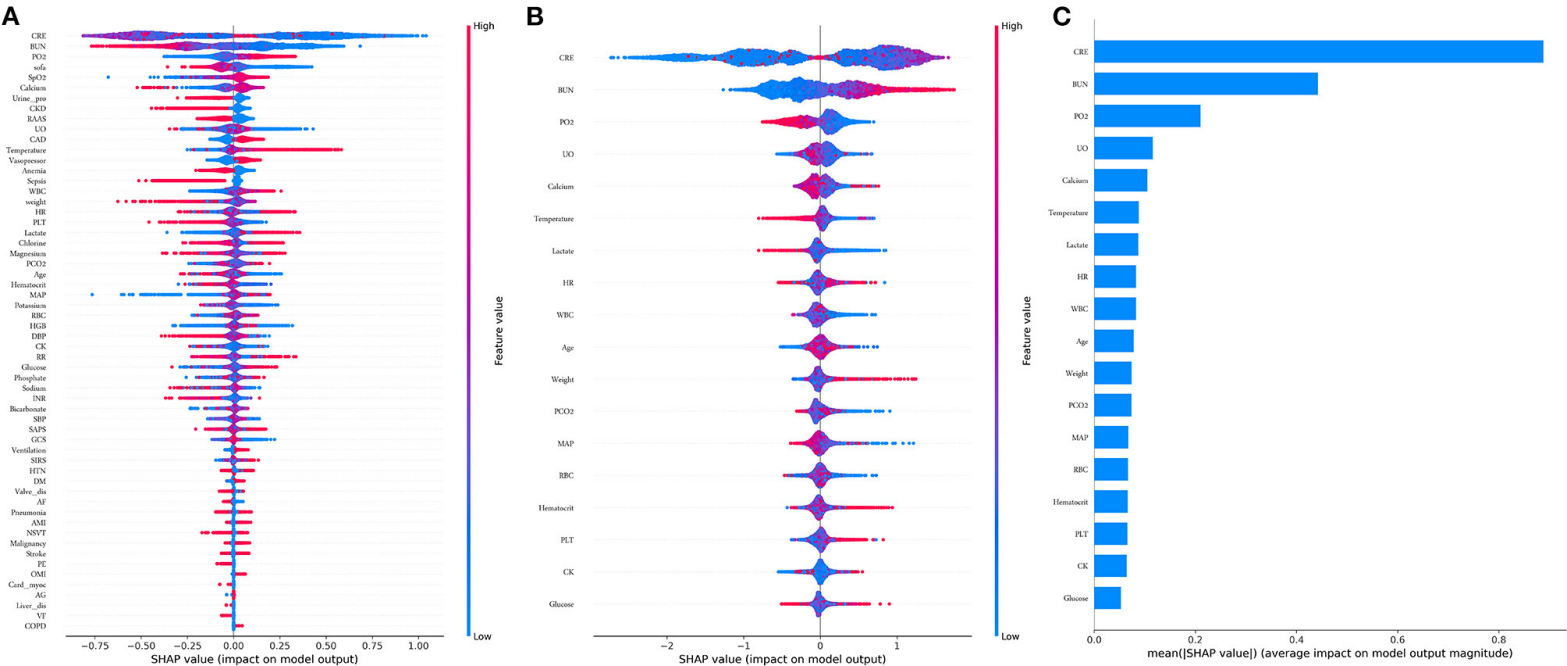
Features importance estimated using the Shapley Additive explanations (SHAP) values. **(A)** All 58 features, the blue to red color represents the feature value (red high, blue low). The x-axis measures the impacts on the model output (right positive, left negative); **(B)** Compact 18 features; **(C)** Significance of the predictors in the LightGBM model. CRE, creatinine; BUN, blood urea nitrogen; PO2, partial pressure of oxygen; UO, urine output; HR, heat rate; WBC, white blood cell; PCO2, partial pressure of carbon dioxygen; MAP, mean aortic pressure. RBC, red blood cell; PLT, platelet; CK, creatine kinase.

Comparisons among 13 ML-based models for the initial prediction of AKI showed that the LightGBM algorithm exhibited the best prediction performance, with an AUROC of 0.803 (Figure 2). Therefore, the LightGBM algorithm was selected as the primary model. Eighteen important features were selected after RFE. The distribution of the effects of each feature in the full and compact models is shown in Figure 3. For the full model with 58 features, the feature importance was evaluated by SHAP value (Figure 3A). Feature importance in the compact model with the 18 selected features is shown in Figures 3B,C. We found that SCr, BUN and PaO_2_ were the three main important risk factors in both the full and compact models.

**Figure 4:**
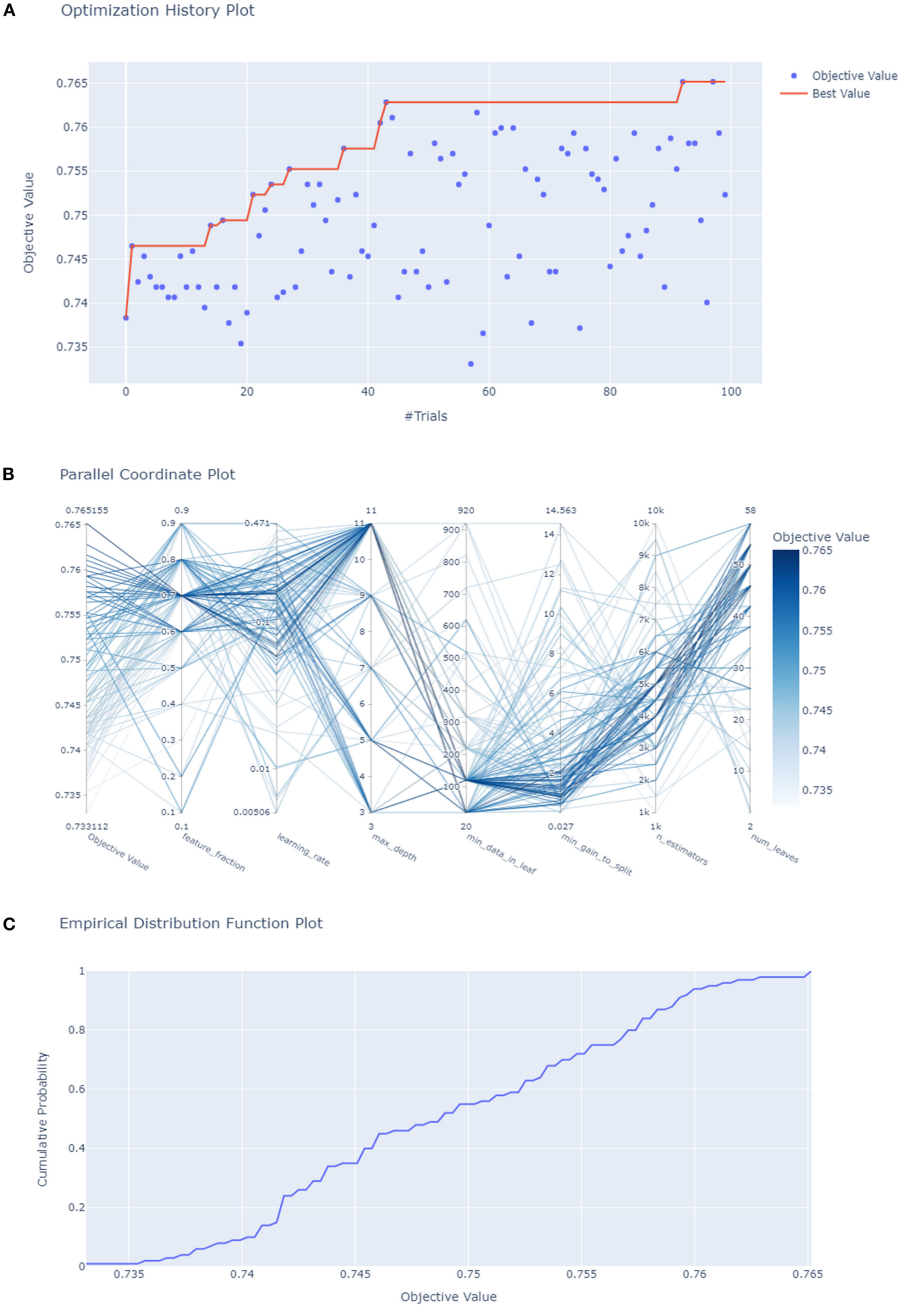
Hyperparameters optimization. **(A)** Each blue point represents the result of a trial, and the dark orange line represents the best AUROC value; **(B)** Each line represents a trial, the shade of color represents the performance of optimization; **(C)** the empirical distribution function of HPO.

**Figure 5:**
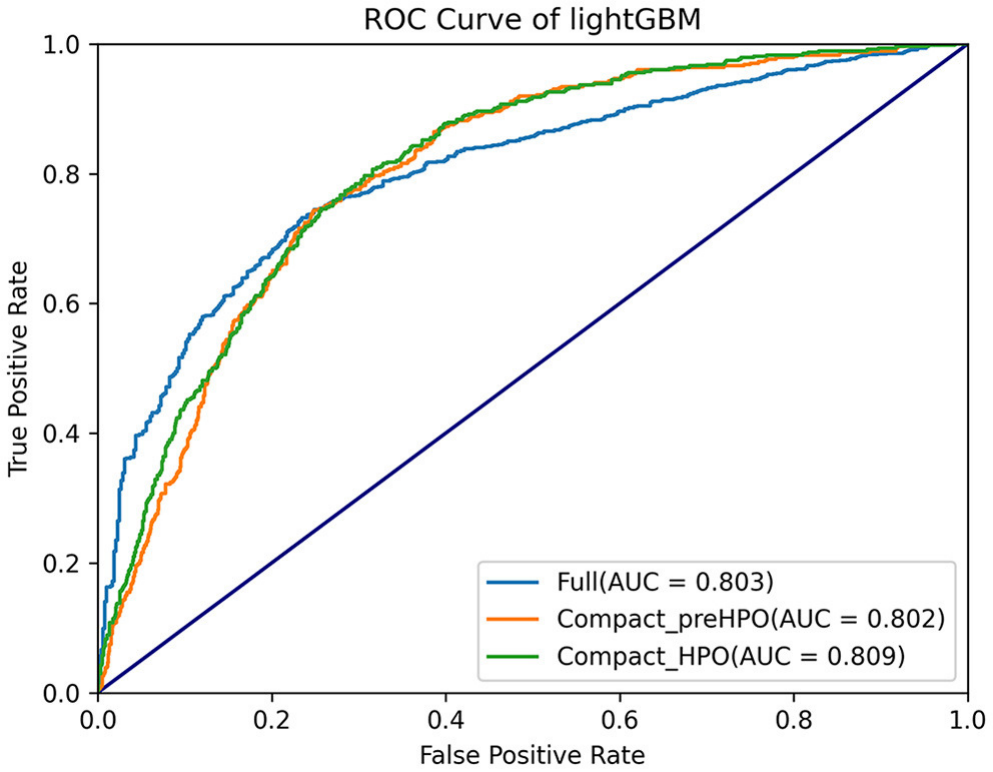
Comparisons of full parameters, pre-HPO and after-HPO compacted parameters models.

Subsequently, HPO was conducted to improve the performance of the compact model (Figure 4). After 100 trials, the LightGBM model with the greatest AUROC was obtained. The final settings of the hyperparameter search are listed in Supplementary Table 2. The performance of a single hyperparameter is shown in Supplementary Figure 1. A comparison was performed between the latest model with the best combination of model parameters and the pre-HPO model in order to confirm the optimization effect (Figure 5). As shown in the figure, the full model had a favorable AUROC of 0.803, and the compact model had a slightly lower prediction performance with an AUROC of 0.802. After HPO, the prediction value of the compact model increased as expected (AUROC = 0.809).

### Model Evaluation and Validation

**Figure 6:**
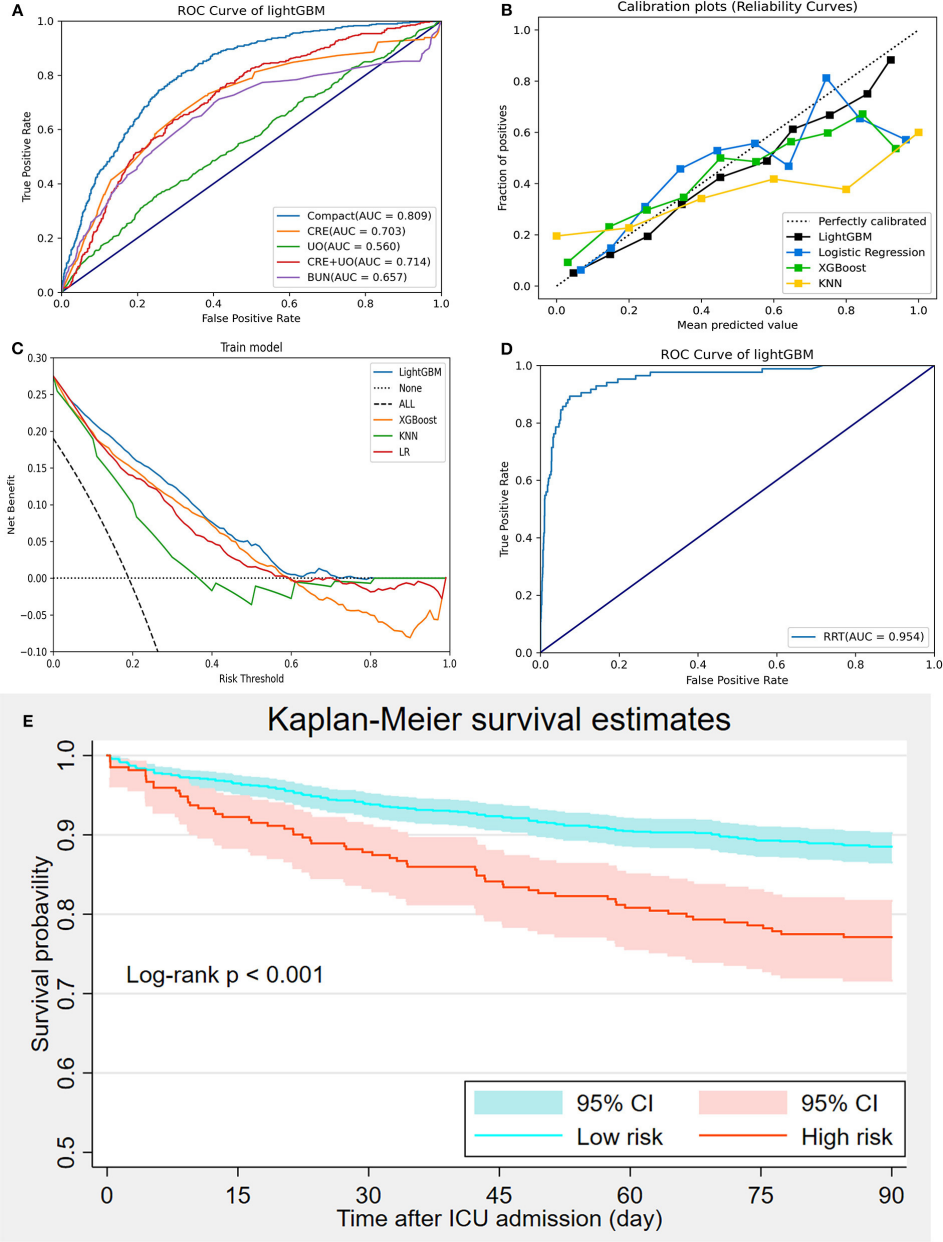
Model evaluation and validation. **(A)** Comparisons of prediction performance between the compact model and measurements of creatinine, urine output, creatinine combined with urine output, and urea nitrogen; **(B)** Calibration curve; **(C)** Decision curve analysis; **(D)** Prediction of renal replacement therapy requirement using the LightGBM model; **(E)** Kaplan-Meier curve analysis of 90-day mortality between high-and low-risk groups divided using the LightGBM.

The AKI prediction performance of the LightGBM model was compared with that of other predictive factors including SCr, UO, BUN, and SCr combined with UO, and we found that the LightGBM model had the best prediction performance (Figure 6A). Other evaluation indicators including AUROC, accuracy, PPV, NPV, BA, F1-score and MCC of the different models based on the 18 features in the internal validation and the external validation sets are summarized in Table 2. As shown in the table, the LightGBM model had the highest AUROC, accuracy and MCC in the two sets. Moreover, it also had the best NPV and BA in the internal validation set of the MIMIC-III database. Calibration curve plotting and DCA were also performed in the present study, and for simplicity, LR, XGBoost and KNN models were selected for comparisons with LightGBM model. As shown in Figure 6B, the prediction probability of the LightGBM model was the closest to the true probability compared with that of the other models. A favorable performance was also observed in the DCA (Figure 6C).

**Table 2:** Model evaluation.

**Model**	**AUROC**	**Accuracy, %**	**PPV, %**	**NPV, %**	**BA, %**	**F1-score**	**MCC**
**Internal validation**							
LightGBM	**0.807**	**76.9**	60.1	**81.6**	**67.9**	0.533	**0.387**
CatBoost	0.795	76.2	60.1	79.7	64.8	0.478	0.344
GBDT	0.798	76.0	60.3	79.4	64.2	0.333	0.336
XGBoost	0.780	75.3	56.3	80.7	66.1	0.414	0.345
Random Forest	0.792	76.3	**61.2**	79.5	64.5	0.326	0.343
LR	0.758	75.1	60.6	77.1	60.0	0.258	0.275
Bagging	0.761	74.2	54.7	78.8	62.7	0.384	0.291
SVM	0.757	72.4	45.5	76.5	56.4	0.351	0.169
Naïve Bayes	0.640	72.7	51.1	75.3	56.3	0.274	0.182
MLP	0.651	69.8	43.3	76.5	58.0	0.456	0.184
KNN	0.608	71.6	42.7	73.5	52.4	0.211	0.089
AdaBoost	0.790	74.9	55.7	80.0	64.9	0.404	0.326
Decision Tree	0.603	69.0	43.8	78.9	61.6	**0.536**	0.230
**External validation**							
LightGBM	**0.816**	**82.8**	59.0	85.0	60.9	0.362	**0.311**
CatBoost	0.791	82.6	**62.4**	83.7	57.5	0.271	0.262
GBDT	0.778	82.1	62.1	82.7	54.2	0.171	0.195
XGBoost	0.790	81.9	52.9	**85.4**	**62.0**	**0.384**	0.303
Random Forest	0.804	82.4	58.7	84.0	58.2	0.293	0.264
Logistic Regression	0.755	82.0	55.9	83.6	57.0	0.261	0.234
Bagging	0.760	81.7	52.1	84.4	59.3	0.325	0.261
SVM	0.707	81.5	46.9	83.6	55.6	0.227	0.185
Naïve Bayes	0.569	73.0	29.2	85.3	58.5	0.322	0.156
MLP	0.692	80.9	47.2	83.6	57.0	0.268	0.207
KNN	0.606	81.4	57.1	81.6	50.9	0.042	0.084
AdaBoost	0.793	82.2	54.4	85.0	61.0	0.363	0.294
Decision Tree	0.622	74.8	34.5	85.3	61.1	0.367	0.212

*The bold values represent the best predict performance among these models. AUROC, the area under the receiver operating characteristic curve; PPV, positive prediction value; NPV, negative prediction value; BA, balanced accuracy; MCC, Matthews correlation coefficient*.

Because many patients with severe AKI have to receive RRT for hyperkalemia, pulmonary edema or anuria, we performed another prediction of RRT requirement during ICU admission based on the LightGBM model using the 18 selected features to further elucidate the prediction performance of the LightGBM model. Figure 6D showed that the performance of the LightGBM model in predicting RRT requirement was satisfactory, with an AUROC of 0.954. In addition, patients from the internal validation set were divided into high- and low-risk groups, as previously described. The comparison of 90-day mortality between the high- and low-risk groups is shown in Figure 6E; the mortality in the high-risk group was significantly higher than that in the low-risk group (log-rank *p* < 0.001).

**Figure 7:**
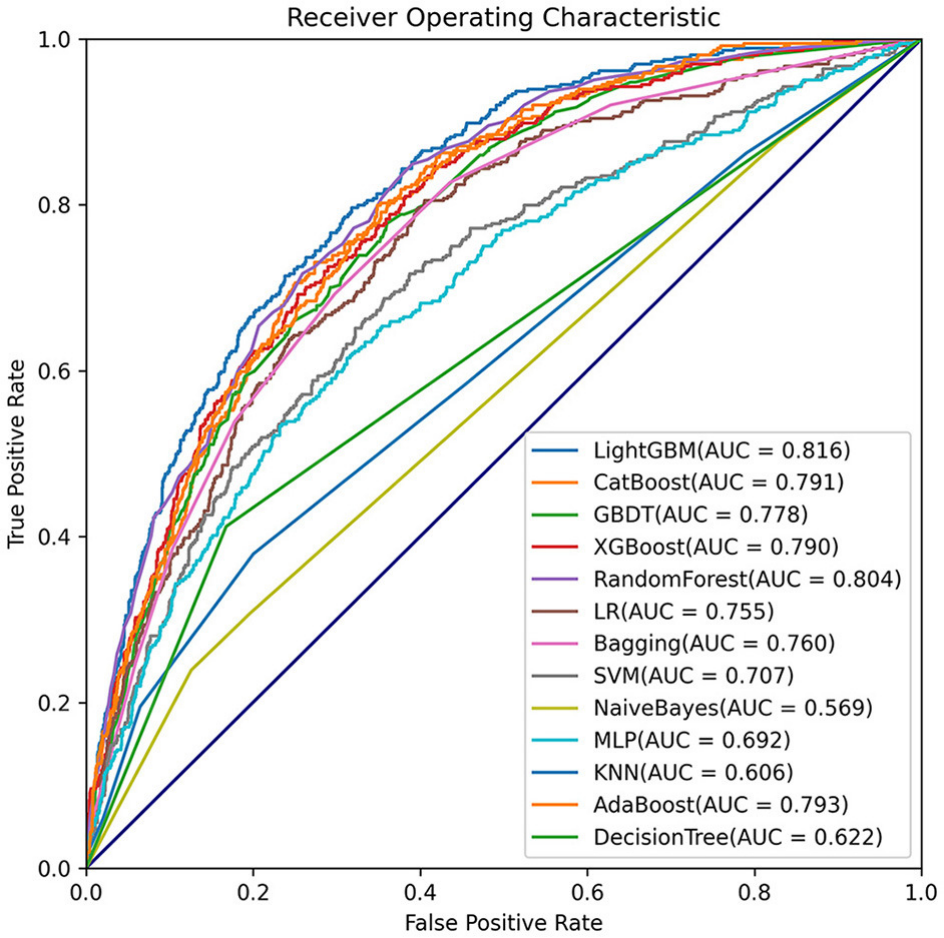
Comparisons of different machine learning models in external validation set.

**Figure 8:**
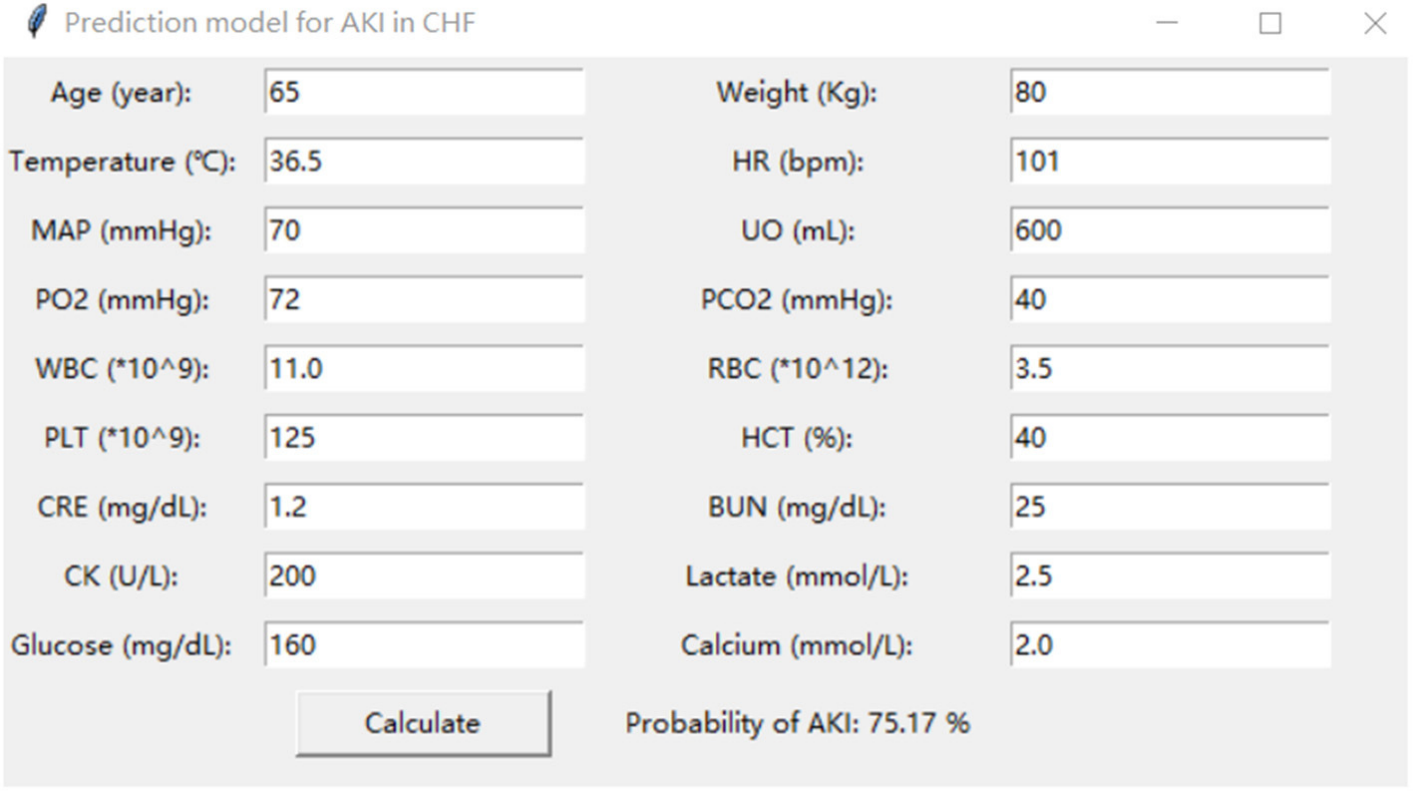
An example of the prediction software.

Moreover, the eICU database was used for external validation of the model. As shown in Figure 7, the LightGBM model had the best prediction performance (AUROC = 0.816) compared with the other models. The accuracy of the LightGBM model in the external validation set was also the highest among the models, and the other evaluation indicators are summarized in Table 2. Finally, we developed a software program based on the 18 features for predicting AKI and determining the probability (Figure 8). The main codes of this study were available at gitee (https://gitee.com/lile_xj/prediction-model-for-aki-in-patients-with-chf).

## Discussion

In the present study, we established a prediction model based on several ML algorithms, and found that various features are strongly associated with AKI in patients with CHF. Among the 13 models, the LightGBM model had the best prediction performance in both the internal and external validation sets. Using this model as the primary model, we found that SCr, BUN and UO were significant risk factors for AKI which is consistent with previous findings (1, 8). Moreover, demographics, such as age and weight; vital signs including temperature, HR and MAP; and various laboratory values, including PaO_2_, PaCO_2_, WBC, RBC, PLT, hematocrit, CK, glucose, blood calcium and lactate were also associated with AKI. When evaluated in both the internal and external validation sets, the LightGBM model also exhibited a favorable performance, predicting RRT requirement during ICU admission and in assessing the short-term mortality in high- and low-risk groups for AKI based on its prediction.

LightGBM is a type of modified gradient boosting algorithm which overcomes the unsatisfactory efficiency and scalability of traditional gradient boosting algorithms, such as XGBoost (22). Several studies have demonstrated that it has a favorable prediction value in the field of medicine (23–25). In this study, we found that LightGBM had the best prediction value compared with XGBoost, LR, naïve Bayes and etc. models. Using the exclusive feature bundling (EFB) method, LightGBM can speed up the training process of the XGBoost algorithm by up to over 20 times while achieving almost the same accuracy (22). Therefore, we suggest that LightGBM, a highly efficient and accurate new ML algorithm, to build some convincing prediction models.

An increase in SCr and a decrease in UO over a short period of time were the main features of AKI and were used to define AKI according to the guidelines (8, 26). BUN is also a classic biomarker for evaluating renal function (27). As mentioned above, SCr, UO, and BUN were the key predictive factors for AKI; however, these biomarkers were not sensitive enough, especially for the early diagnosis of AKI (28). Accordingly, we compared the prediction performance between the compact model and measurements of SCr, UO, SCr combined with UO, and BUN and found that the performance of the compact model was significantly better than the that of these measurements (AUROCs: 0.809, 0.703, 0.560, 0.714 and 0.675, respectively) (Figure 6A). In addition, although chronic kidney disease (CKD) is an important risk factor for AKI (6), it was excluded in the compact model through RFE. The reason may be that the participants with CKD were not divided into different groups based on the severity of renal dysfunction in the present study; stage 1 or 2 CKD may be less strongly associated with AKI than moderate to severe CKD (29). Future studies are required to address this issue.

Furthermore, we found that elderly and obese patients may at a high risk of AKI, which was consistent with the findings of previous studies (30, 31). Abnormal vital signs, including temperature, HR, and MAP, were associated with AKI, as expected. Other laboratory values, including PaO_2_, PaCO_2_, WBC, RBC, PLT, hematocrit, CK, glucose, calcium, and lactate, which are not commonly used in prediction models, were also found to be predictive factors for AKI; this information would be helpful in future research (Figure 3B).

To further evaluate the prediction performance, the LightGBM model was used to predict RRT requirement during ICU admission using the 18 selected features. AKI is strongly associated with increased early and long-term mortality (32, 33), and some patients with severe AKI must receive RRT for hyperkalemia, pulmonary edema or anuria (34). In the present study, the LightGBM model was also able to efficiently predict RRT requirement with an AUROC of 0.954, which again demonstrated the satisfactory prediction performance of LightGBM. Moreover, all patients in the internal validation set were divided into high- and low-risk groups for AKI using the model, and we performed a Kaplan-Meier curve analysis to compare the 90-day mortality between the two groups. The result showed that the 90-day mortality in the high-risk group was significantly higher than that in the low-risk group (log-rank *p* < 0.001). In addition, the LightGBM model also had the best prediction value in the external validation set from the eICU database, indicating a remarkable ability for generalization and clinical value. The favorable prediction performance of the LightGBM model was demonstrated in various aspects, suggesting its clinical application value in the early identification of AKI in patients with CHF and consequently in the administration of appropriate preventive treatments.

## Strengths and Limitations

Our study, which was based on two large-scale cohorts, has contributed to establishing a prediction model for AKI. To our knowledge, the present study has built the first ML-based prediction model for AKI in patients with CHF. Furthermore, a series of ML algorithms were screened to select the best prediction model and guarantee the satisfactory prediction performance. Moreover, RFE was performed to identify the important prediction features and to exclude weakly correlated factors, which also improved the clinical feasibility. Additionally, HPO could improve the prediction performance of the ML-based model. Finally, a large cohort was included in this study as the external validation set, and internal and the external validation enhanced the reliability of the model.

There were some limitations associated with this study. First, we collected related data from two public databases, and some features, including B-type natriuretic peptide and cardiac troponin I levels were excluded because of the high rate of missing data. Feature selection is an important aspect of building a prediction model, and excluding certain variables might affect the prediction performance. Second, the participants were all from the ICU, where patients are more likely to have multiple-organ dysfunction and poor prognosis compared with those in general wards, which may limit the target population of the prediction model. Finally, this study was based on a retrospective analysis of data and the results should be confirmed through further prospective studies.

## Conclusion

In conclusion, we established a prediction model based on ML algorithms, which included 18 clinical features, and found that the LightGBM model could predict AKI in CHF patients with high accuracy and that the prediction performance was better than that of other clinical models. Moreover, the model may help in predicting RRT requirement and identifying the population of patients with poor prognosis among those with CHF. These findings need to be confirmed in future prospective studies.

## Data Availability Statement

Publicly available datasets were analyzed in this study. This data can be found here: https://mimic.mit.edu/.

## Ethics Statement

Ethical review and approval was not required for the study on human participants in accordance with the local legislation and institutional requirements. Written informed consent for participation was not required for this study in accordance with the national legislation and the institutional requirements.

## Author Contributions

This study was designed by LL and XP. XYW was responsible for machine learning analysis. LL were responsible for data collation and statistical analysis. XP and LL wrote the first draft. HZ reviewed and checked the manuscript. All authors read and approved the final manuscript. All authors contributed to the article and approved the submitted version.

## Funding

This project was supported by the Beijing Hospital Clinical Research 121 Project (BJ-2018-201).

## Conflict of Interest

The authors declare that the research was conducted in the absence of any commercial or financial relationships that could be construed as a potential conflict of interest.

## Publisher's Note

All claims expressed in this article are solely those of the authors and do not necessarily represent those of their affiliated organizations, or those of the publisher, the editors and the reviewers. Any product that may be evaluated in this article, or claim that may be made by its manufacturer, is not guaranteed or endorsed by the publisher.
