# Supplementary material for: A Machine Learning-Based Prediction Model for Acute Kidney Injury in Patients With Congestive Heart Failure
Source: Front Cardiovasc Med. 2022 Mar 4;9:842873. doi: 10.3389/fcvm.2022.842873 (PMC8931220; doi:10.3389/fcvm.2022.842873)
Supplement: Supplementary file 1 [file Data_Sheet_1.docx]

| Table S1. Baseline characteristics of all features. | | | |
| --- | --- | --- | --- |
| Variables | Non-AKI  (n = 6216) | AKI  (n = 2364) | *P* value |
| Demographics and characteristics |  |  |  |
| Age, years | 72.3 ± 13.7 | 75.0 ± 12.7 | < 0.001 |
| Weight, kg, | 80.6 ± 22.3 | 82.6 ± 24.8 | < 0.001 |
| SOFA score | 4.49 ± 3.00 | 5.47 ± 3.05 | < 0.001 |
| SAPS-II | 19.1 ± 5.2 | 20.5 ± 5.1 | < 0.001 |
| SIRS score | 2.74 ± 1.01 | 2.75 ± 0.98 | 0.630 |
| GCS score | 13.7 ± 2.7 | 13.6 ± 2.6 | 0.110 |
| Vital signs |  |  |  |
| Temperature, ℃ | 36.8 ± 0.6 | 36.6 ± 0.7 | < 0.001 |
| RR, bpm | 19.4 ± 4.1 | 20.1 ± 4.1 | < 0.001 |
| HR, bpm | 102.9 ± 21.2 | 102.1 ± 22.6 | 0.145 |
| SBP, mmHg | 116.7 ± 16.8 | 115.3 ± 17.7 | 0.001 |
| DBP, mmHg | 57.7 ± 10.2 | 56.9 ± 10.6 | 0.001 |
| MAP, mmHg | 75.7 ± 10.5 | 73.8 ± 10.8 | < 0.001 |
| SpO_2_, % | 96.9 ± 2.8 | 96.4 ± 3.2 | < 0.001 |
| UO, mL | 1813 ± 1171 | 1639 ± 1193 | < 0.001 |
| Therapy |  |  |  |
| Mechanical ventilation use (1st 24 h) | 3019 (48.6) | 897 (37.9) | < 0.001 |
| Vasopressor use (1st 24 h) | 2736 (44.0) | 932 (39.4) | < 0.001 |
| ACEI/ARB use | 2299 (37.0) | 890 (37.6) | 0.570 |
| Comorbidities |  |  |  |
| Angina pectoris, % | 2807 (45.2) | 883 (37.4) | < 0.001 |
| AMI, % | 573 (9.2) | 161 (6.8) | < 0.001 |
| OMI, % | 811 (13.0) | 303 (12.8) | 0.777 |
| AF, % | 2699 (43.4) | 1094 (46.3) | 0.017 |
| NSVT, % | 477 (7.7) | 179 (7.6) | 0.874 |
| VF, % | 156 (2.5) | 44 (1.9) | 0.075 |
| Cardiomyopathy, % | 91 (1.5) | 45 (1.9) | 0.145 |
| Valvular disease, % | 2069 (33.3) | 684 (28.9) | < 0.001 |
| PE, % | 148 (2.4) | 72 (3.0) | 0.082 |
| Hypertension, % | 2695 (43.4) | 760 (32.1) | < 0.001 |
| Diabetes, % | 2168 (34.9) | 956 (40.4) | < 0.001 |
| Pneumonia, % | 1495 (24.1) | 714 (30.2) | < 0.001 |
| COPD, % | 241 (3.9) | 115 (4.9) | 0.040 |
| CKD, % | 476 (7.7) | 541 (22.9) | < 0.001 |
| Sepsis, % | 480 (7.7) | 392 (16.6) | < 0.001 |
| Liver disease, % | 274 (4.4) | 130 (5.5) | 0.033 |
| Stroke, % | 376 (6.0) | 106 (4.5) | 0.005 |
| Cancer, % | 1063 (17.1) | 437 (18.5) | 0.131 |
| Anemia, % | 2232 (35.9) | 1063 (45.0) | < 0.001 |
| Urine protein, % | 1288 (20.7) | 807 (34.1) | < 0.001 |
| Laboratory tests |  |  |  |
| PaO_2_, mmHg | 169.2 ± 88.7 | 137.7 ± 74.7 | < 0.001 |
| PaCO_2_, mmHg | 44.5 ± 12.7 | 44.1 ± 13.4 | 0.247 |
| WBC, × 10^9/L | 12.2 ± 6.1 | 12.2 ± 6.5 | 0.618 |
| RBC, × 10^12/L | 3.57 ± 0.68 | 3.55 ± 0.70 | 0.138 |
| Hemoglobin, g/dL | 10.7 ± 2.1 | 10.5 ± 2.0 | 0.002 |
| Hematocrit, % | 32.0 ± 6.1 | 31.9 ± 6.1 | 0.228 |
| Platelet, × 10^9/L | 216.2 ± 100.2 | 221.9 ± 105.1 | 0.024 |
| SCr, mg/dL | 1.47 ± 1.55 | 2.04 ± 1.33 | < 0.001 |
| BUN, mg/dL | 28.0 ± 19.9 | 46.1 ± 26.7 | < 0.001 |
| CK, U/L | 467 ± 900 | 442 ± 926 | 0.246 |
| Lactate, mmol/L | 2.21 ± 1.52 | 2.22 ± 1.62 | 0.832 |
| Glucose, mg/dL | 141.8 ± 44.2 | 145.8 ± 48.8 | < 0.001 |
| Sodium, mmol/L | 138.0 ± 4.7 | 138.5 ± 5.4 | < 0.001 |
| Potassium, mmol/L | 4.24 ± 0.77 | 4.38 ± 0.80 | < 0.001 |
| Anion gap, mmol/L | 9.89 ± 0.45 | 9.96 ± 0.28 | < 0.001 |
| Bicarbonate, mmol/L | 24.5 ± 4.7 | 23.6 ± 5.3 | < 0.001 |
| Chlorine, mmol/L | 104.2 ± 6.2 | 104.0 ± 6.7 | 0.136 |
| Phosphate, mg/dL | 3.73 ± 1.27 | 4.20 ± 1.40 | < 0.001 |
| Magnesium, mg/dL | 2.01 ± 0.40 | 2.09 ± 0.42 | < 0.001 |
| Calcium, mmol/L | 8.38 ± 0.84 | 8.37 ± 0.88 | 0.542 |
| INR | 1.80 ± 1.55 | 2.05 ± 2.18 | < 0.001 |

AKI: acute kidney injury; SOFA: sequential organ failure assessment; SAPS: simplified acute physiology score; SIRS: systemic inflammatory response syndrome; GCS: glasgow coma scale; RR: respiratory rate; HR: heart rate; SBP: systolic blood pressure; DBP: diastolic blood pressure; SpO2: saturation of pulse oxygen; MAP: mean aortic pressure; UO: urine output; ACEI: angiotensin-converting enzyme inhibitors; ARB: angiotensin receptor blockers; AMI: acute myocardial infarction; OMI: old myocardial infarction; AF: atrial fibrillation; NSVT: non-supraventricular tachycardia; VF: ventricular fibrillation; PE: pulmonary embolism; COPD: chronic obstructive pulmonary disease; CKD: chronic kidney disease; PaO2: arterial partial pressure of carbon dioxide; WBC: white blood cell count; RBC: red blood cell count; SCr: Serum creatinine; BUN: blood urine nitrogen, CK: creatine kinase; INR: international normalized ratio.

| **Table S2**. Hyperparameter search domains and final settings | | |
| --- | --- | --- |
| Hyperparameters | Search domain | Final setting |
| 'n_estimators' | [1000, 10000] | 4000 |
| 'max_depth' | [3, 11] | 11 |
| 'num_leaves' | [2, 58] | 54 |
| 'learning_rate' | [0.01, 0.5] | 0.1635 |
| 'min_gain_to_split' | [0, 15] | 0.8030 |
| 'min_data_in_leaf' | [20, 1000] | 120 |
| 'feature_fraction' | [0.1, 0.9] | 0.7 |

**
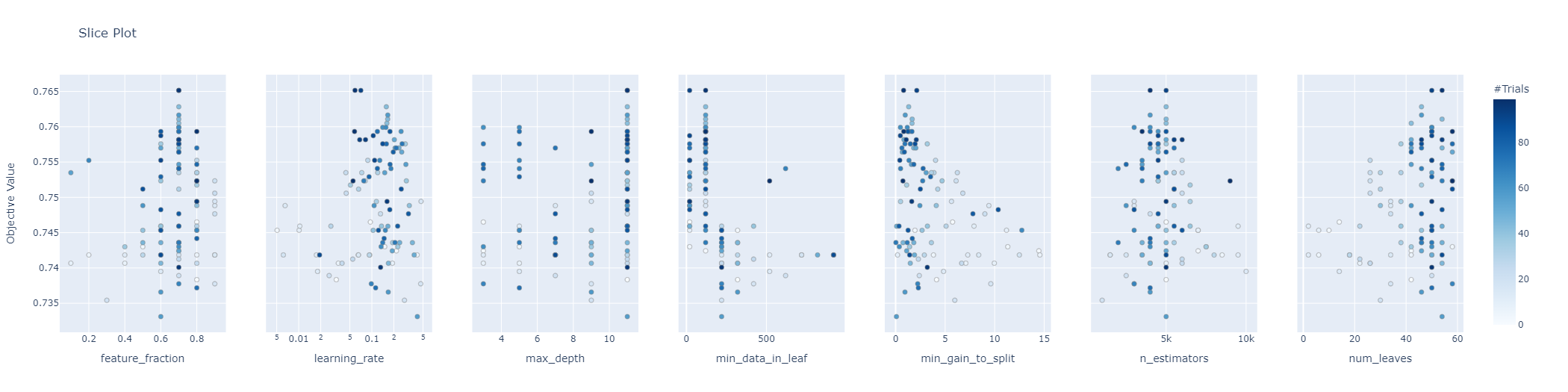
Figure S1**. Performance of single hyperparameter by slice plot of hyperparameters optimization
